# Coaction of Spheroid-Derived Stem-Like Cells and Endothelial Progenitor Cells Promotes Development of Colon Cancer

**DOI:** 10.1371/journal.pone.0039069

**Published:** 2012-06-26

**Authors:** Bo Wei, Xiao-Yan Han, Cui-Ling Qi, Shi Zhang, Zong-Heng Zheng, Yong Huang, Tu-Feng Chen, Hong-Bo Wei

**Affiliations:** 1 Department of Gastrointestinal Surgery, the Third Affiliated Hospital of Sun Yat-sen University, Guangzhou, China; 2 Central Laboratory, the Third Affiliated Hospital of Sun Yat-sen University, Guangzhou, China; 3 Institute of Vascular Biology, Guangdong Pharmaceutical University, Guangzhou, China; 4 Department of Surgery, the University of Hong Kong, Hong Kong; University of Torino, Italy

## Abstract

Although some studies described the characteristics of colon cancer stem cells (CSCs) and the role of endothelial progenitor cells (EPCs) in neovascularization, it is still controversial whether an interaction exists or not between CSCs and EPCs. In the present study, HCT116 and HT29 sphere models, which are known to be the cells enriching CSCs, were established to investigate the roles of this interaction in development and metastasis of colon cancer. Compared with their parental counterparts, spheroid cells demonstrated higher capacity of invasion, higher tumorigenic and metastatic potential. Then the in vitro and in vivo relationship between CSCs and EPCs were studied by using capillary tube formation assay and xenograft models. Our results showed that spheroid cells could promote the proliferation, migration and tube formation of EPCs through secretion of vascular endothelial growth factor (VEGF). Meanwhile, the EPCs could increase tumorigenic capacity of spheroid cells through angiogenesis. Furthermore, higher microvessel density was detected in the area enriching cancer stem cells in human colon cancer tissue. Our findings indicate that spheroid cells possess the characteristics of cancer stem cells, and the coaction of CSCs and EPCs may play an important role in the development of colon cancer.

## Introduction

Colorectal cancer is the third leading cause of cancer deaths worldwide, and the 5-year relative survival rate is only 53.8–65.2% despite diagnostic and therapeutic advances [1], [2]. Tumor recurrence and metastasis are two critical survival-influencing factors of colorectal cancer.

Recently, increasing evidence suggests that tumor initiation and metastases are dependent on a small sub-population of tumor cells termed cancer stem cells (CSCs) bearing infinite self-renewal potential and the capacity to differentiate into diverse populations comprising a tumor [3]. According to this model, cancer stem cells were found to sustain carcinogenesis, metastasis, and recurrence of colorectal cancer.

The existence of cancer stem cells was first proven in the context of acute myeloid leukemia [4]. This principle was further extended to some solid tumors, including colon cancer, breast cancer, brain tumors, and lung cancer [5], [6], [7], [8], [9], [10]. Cell sorting of a sub-population on the basis of cell surface markers and confirmation of their tumor-initiating activity in xenograft transplantation assays are commonly used procedures for isolation and identification of CSCs from tumor tissues or cell lines [11]. Although CD133, CD44, EpCAM were used extensively as cell surface markers for colon cancer stem cells, there were still doubts on these cell surface markers [12], [13]. Side population (SP) cells isolation were once used to enrich the cancer stem cells. But several studies showed evidence against an association between the SP cells and cancer stem cells [14], [15], [16], [17]. Recently, increasing researches have reported that the non-adherent, three-dimensional (3D) tumor spheres under serum-free conditions could efficiently enrich cancer stem cells [18], [19], [20] *in vitro*. Therefore, this method was applied to enrich colon cancer stem cells in the present study.

Endothelial progenitor cells (EPCs), a minor subpopulation of the mononuclear cell fraction in peripheral blood, originate mainly from the bone marrow-derived cells with the ability to differentiate into mature endothelial cells [21], [22]. EPCs leave the bone marrow and recruit to sites requiring vascular repair following gradients of growth factors and cytokines that are released into the circulation by injured tissues or tumors, and then contribute to blood vessel formation [23], [24]. Recent evidences demonstrated that circulating EPCs play an important role in tumor neovascularization, tumor growth, and metastasis [25], [26], [27], [28], [29].

Although the contribution of EPCs to tumor neovascularization has been demonstrated in several types of cancer, the interaction between EPCs and colon cancer stem cells have not been reported. The aim of this study was to determine the bionomics of colon cancer stem cells and the role of coaction of CSCs and EPCs in the growth and metastasis of colon cancer.

## Materials and Methods

### Ethics Statement

All experiments involving human participants (including collection of human umbilical cord blood and colon cancer samples) have been approved by the Medical Research Ethics Committee of Sun Yat-sen University, and conducted according to the principles expressed in the Declaration of Helsinki. All participants involved in the study signed the informed consent forms and all animal experiments were conducted according to relevant national and international guidelines. And this project was approved by the Medical Research Animal Ethics Committee of Sun Yat-sen University.

### Cell Culture

HCT116 (ATCC, CCL-247) and HT29 (ATCC, HTB-38) colon cancer cell lines were maintained in DMEM/F12 supplemented with 10% FBS, 200 U/ml penicillin, 200 µg/ml streptomycin. Tumorsphere media (also called as serum free medium, SFM) was composed of DMEM/F12 media supplemented with 1×B27 (Invitrogen), EGF (20 ng/ml, Peprotech), bFGF (10 ng/ml, Peprotech), routine insulin (5 µg/ml, Invitrogen), 200 U/ml penicillin, and 200 µg/ml streptomycin. For 3D suspension culture, HCT116 and HT29 cells grown in two dimensional monolayer were digested with trypsin, resuspended, and then seeded at a density of 2×10^6^ cells in SFM in 100 mm ultra-low attachment dishes (Corning) at 37°C in a humidified 5% CO2 ? 95% air atmosphere.

### Detection of CD133 Expression by Flow Cytometry

The cells derived from monolayer cultures and suspension spheres on day 7 after primary culture were detected for the expression of CD133. The cells were washed twice in cold PBS, and subsequently cell suspensions were incubated at 4°C with 1∶10 FITC-conjugated mouse monoclonal antihuman CD133 Ab (Miltenyi biotec) for 45 minutes in the dark. After incubation, the cells were washed twice in cold PBS with 1% BSA and resuspended in 400 µl cold PBS with 1% BSA for flow cytometry analysis within 1 hour.

### Self-renew and Multi-differentiation Assay

Spheroid cells were dissociated into single cells and plated in 6-well plates at 100 cells per well in 2 ml SFM. To evaluate the self-renew capacity of spheroid cells, cell immunofluorescence staining of Lgr5 and CK20 were performed after 7 days’ culturing. Cell differentiation was induced by culturing in DMEM/F12 supplemented with 10% FBS in common flasks. Four weeks later, the same cell immunofluorescence staining was performed, and the expression of CD133 was determined by FCM as described before.

### Cell Proliferation and Colony Formation Assay

Single-cell suspensions of spheroid and adherent cells were prepared and seeded at 1×10^4^ cells per well in 96-well plates, and cultured in either SFM or serum-containing media for 24 h. Cell proliferation was performed on day 1, 2, 3, 4, 5, 6, and 7 using Cell Counting Kit-8 (Dojindo, Japan). The plate colony formation assay was carried out to determine the colony formation efficacy of cell in the four groups. Single-cell suspensions were plated at 1×10^2^ cells per well in 6-well plates, and cultured in serum-containing media at 37°C in 5% CO2 for 2 weeks. Then the plates were stained with Giemsa-Wright's stain. The number of colonies within each well was counted and photographed. The experiments were independently performed at least three times.

### Homogeneity and Heterogeneity Adhesion Assay

The adhesion ability of cells to ECM was tested using fibronectin (FN)-coated 96-well plates (Corning). Single-cell suspensions of spheroid and adherent cells were plated (10^5^/well), and incubated at 37°C for 2 h, then washed with PBS to remove the non-adhesive cells. Delt OD of 570 nm wavelength was determined to reflect the FN-adherent cells using Cell Counting Kit-8 (Dojindo, Japan). The adhesion ability of cells to homogeneous cells was tested using monolayer cells-paved 96-well plates. Spheroid and adherent cells were plated at 10^5^ per well, and incubated at 37°C for 60 min, 90 min, and 120 min. Then the non-adhesive cells were counted and homotypic adhesive activity was calculated using the formula: homotypic adhesion (%) = (total cell number - non-adhesive cells)/total cells×100%.

### Migration and Invasion Assays

Migration and invasion assays were performed in 24-well plate transwell chambers with 8 µm-pore polycarbonate filter inserts (Corning). A total of 5×10^4^ or 10^5^ dissociated spheroid or adherent cells were seeded on uncoated or Matrigel-coated inserts in 100 µl of serum-free medium inserts for migration or invasion assays respectively. The lower chambers were filled with 0.5 ml of 10% FBS-supplemented DMEM/F12 medium. After 24 h and/or 48 h, cells on the upper side of the filter were removed and the cells on the lower surface of the insert were fixed and stained with crystal violet. The number of stained cells was counted under a light microscope. Assays were performed in triplicates.

### Western Blot Analysis

The total cell extracts were separated by 10% SDS-polyacrylamide gel electrophoresis and transferred to Polyvinylidene Fluoride Membrane (Millipore) for ICAM-1, CXCR4 and VEGF detection. The membranes were washed in Tris-buffered saline with Tween (TBST, composed of 10 mM Tris-HCl, pH 8, 150 mM NaCl, and 0.05% Tween 20), blocked 1 h at room temperature with 5% nonfat milk in TBST, and then probed 1 h at room temperature with anti-ICAM-1 (Santa Cruz Biotechnologies), anti-CXCR4 (Abcam), anti-VEGF (BD Pharmingen) and anti-GAPDH (Peprotech). After incubation with horseradish peroxide-conjugated secondary antibody, immunoreactive proteins were detected by ECL detection system (Millipore). Protein concentrations were determined by using the BCA protein assay kit (ThermoScientific Biosciences).

### Isolation and Assessment of EPCs

Mononuclear cells were isolated by density gradient centrifugation from human umbilical cord blood and cultured in fibronectin-coated flask using M199 supplemented with 20% FBS and 1% extracts of mouse cerebral tissue. EPCs were purified basing on different adherence rate, and non-adherent cells within 24 h were transferred to another fibronectin-coated flask. After 14 days’ culturing, EPCs were incubated with PE-CD133, FITC-CD34, PE-VEGFR2 or isotype-matched IgG controls and identified by flow cytometry as described before.

### Cell Proliferation and Migration of EPCs

Single-cell suspension of EPCs was seeded at 2×10^4^ cells per well in 96-well plates, and cultured in M199 containing 0, 20%, and 40% supernatant of spheroid or adherent cells. Cell proliferation was performed on day 1, 2, 3, 4, 5, 6, and 7 using Cell Counting Kit-8 (Dojindo, Japan). Migration assays were performed to evaluate recruitment of EPCs by spheroid or adherent cells. Single-cell suspension of EPCs was inoculated at 5×10^4^ cells per well on inserts in 100 µl of serum-free medium. The lower chambers were filled with 0.5 ml of DMEM/F12 containing 40% supernatant of spheroid or adherent cells. After 24 h, migrated cells were counted as describe before. Assays were performed in triplicates.

### *In vitro* Capillary Tube Formation

Supernatants of HT29, HT29 Sphere, HCT116 and HCT116 Sphere were added to 24-well plates (Corning Inc) coated with growth factor reduced Matrigel (BD Biosciences). To illustrate if tube formation capacity of EPCs can be influenced by blocking VEGF, bevacizumab (avastin, a VEGF monoclone antibody, Genentech Inc) was added to supernatant of spheroid cells at a concentration of 0.25 mg/ml as another two groups [30], [31]. Then a total of 5×10^4^ EPCs were seeded and incubated at 37°C, 5% CO2 for 3 to 30 h. After 24 h, capillary tubes were counted in random fields from each well. Assays were performed in triplicates.

### ELISA

To demonstrate the a paracrine effect of cancer stem cells and confirm the result of VEGF detection from Westerm Blot analysis, we investigated VEGF secretion by cancer stem cells using a quantitative method. VEGF concentration in supernatant of spheroid/adherent cells were determined by ELISA (R&D Systems) according to the protocol provided by the manufacturer. And also, the experiment was performed three times.

### *In vivo* Tumorigenesis

To determine whether spheroid cells are more tumorigenic than their adherent counterparts *in vivo* and whether EPCs promote their tumorigenic capacity by increasing tumor angiogenesis, we carried out tumor development and liver metastasis experiments. Single cells were resuspended in PBS. And then 100 µl suspensions containing 2×10^6^ adherent cells, spheroid cells, or spheroid cells plus 2×10^5^ or 2×10^4^ EPCs (the spheroid cells and EPCs ratio of 10∶1 and 100∶1) were injected subcutaneously (s.c.) into the flanks of 4- to 6-week-old male BALB/C-nu mouse, obtained from the Jackson Laboratory. Subcutaneous tumor diameters were measured with a digital caliper every two or three days, and tumor volume in mm^3^ was calculated using the formula: Volume  =  width^2^×length×0.52. Then 3 or 4 weeks later, the mice were killed and frozen tissue sections (6 µm) of subcutaneous tumors were made for immunofluorescent staining to observe the angiogenesis in each group. Here we use a non-specific polyclonal CD31 antibody to detect the neovascularization of the subcutaneous tumor, and another monoclonal human-specific CD31 antibody for the EPCs contribution to angiogoiesis.

### *In vivo* Liver Metastasis

Liver metastatic model was used to determine the metastatic potential of spheroid cells and whether EPCs promote this capacity by increasing tumor angiogenesis. Hepatic metastases were produced by intrasplenic injection of 4×10^6^ adherent cells, spheroid cells, or speroid cells plus 4×10^5^ or 2×10^4^ EPCs (the ratio of 10∶1 or 100∶1), and then splenectomy was performed 10 min after intrasplenic injection to avoid splend metastasis. The overall health condition was recorded in detail once a day. Liver metastasis foci were examined and measured. And tissue section was made for HE staining to confirm the pathological source.

### Clinical Evidence Obtained by Immunofluorescent Staining

Frozen sections (6 µm) of colon cancer tissue and normal colon mucosa (n = 15, from the same patient) were fixed with 4% paraformaldehyde/PBS, treated with microwave for 10 min for immunofluorescent staining. The anti-CD31 Ab (Fisher Scientific), the anti-CD133 Ab (Abcam), the anti-Lgr5 Ab (Abcam), and the anti-CK20 Ab (Santa Cruz Biotechnologies) were used according to the manufacturer’s protocol. Counterstaining of nuclei with DAPI (Invitrogen) was also performed. The sections were incubated with according secondary DeLight448- or 555-conjugated anti-mouse or rabbit Ab (Abcam) for 1 h at room temperature or overnight at 4°C. Images were captured with a Zeiss confocal microscope (LSM-710). Here, throughout this manuscript, data from at least three independent experiments have been analyzed to verify reproducibility of results.

### Statistical Analysis

Statistical significance was determined by one-way ANOVA followed by Bonferroni post-hoc test for multiple comparisons or Student’s t-test. For all tests, P<0.05 or <0.01 was considered significant or highly significant statistically.

## Results

### Generation and Characterization of Colonospheres

**Figure 1:**
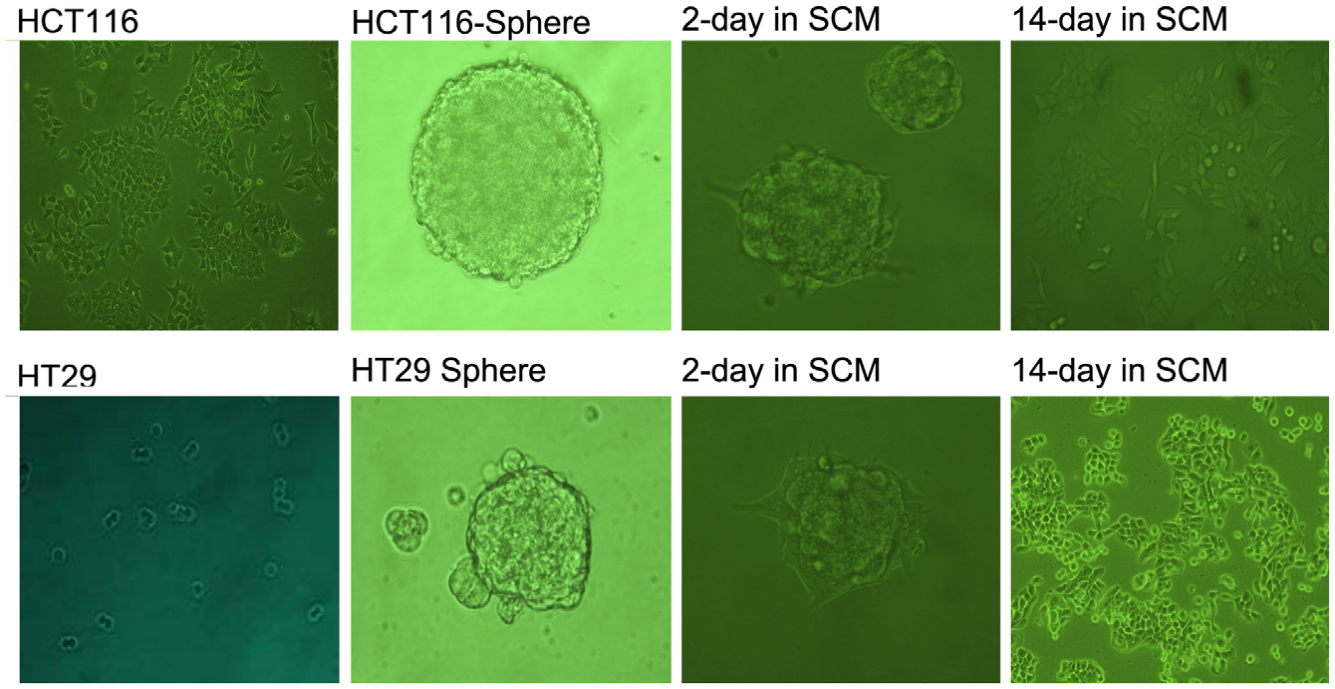
Morphological change of adherent, spheres, and differentiated HCT-116 and HT29. Both HCT116 and HT29 can form large round, unattached floating colonsphere of 50–100 µm when cultured in SFM. When spheroid cells were cultured in 10% FBS containing medium, the floating spheroid cells attached to the plastic, gradually migrated from colonspheres and transformed to adherent cells.

Both HCT116 and HT29 colon cancer cells grew in large round, unattached floating spheroid colonies (termed colonospheres) when they were cultured in serum free medium supplemented with 1×B27, 20 ng/ml EGF, 10 ng/ml bFGF (Figure 1). The frequency of sphere-forming cells was determined by performing an extreme limiting dilution analysis (ELDA) for parental and colonospheres-derived HCT-116 cells. The frequency to form spheres was found to be 5.5 fold higher for cells derived from colonospheres than those from the parental cell lines.

**Figure 2:**
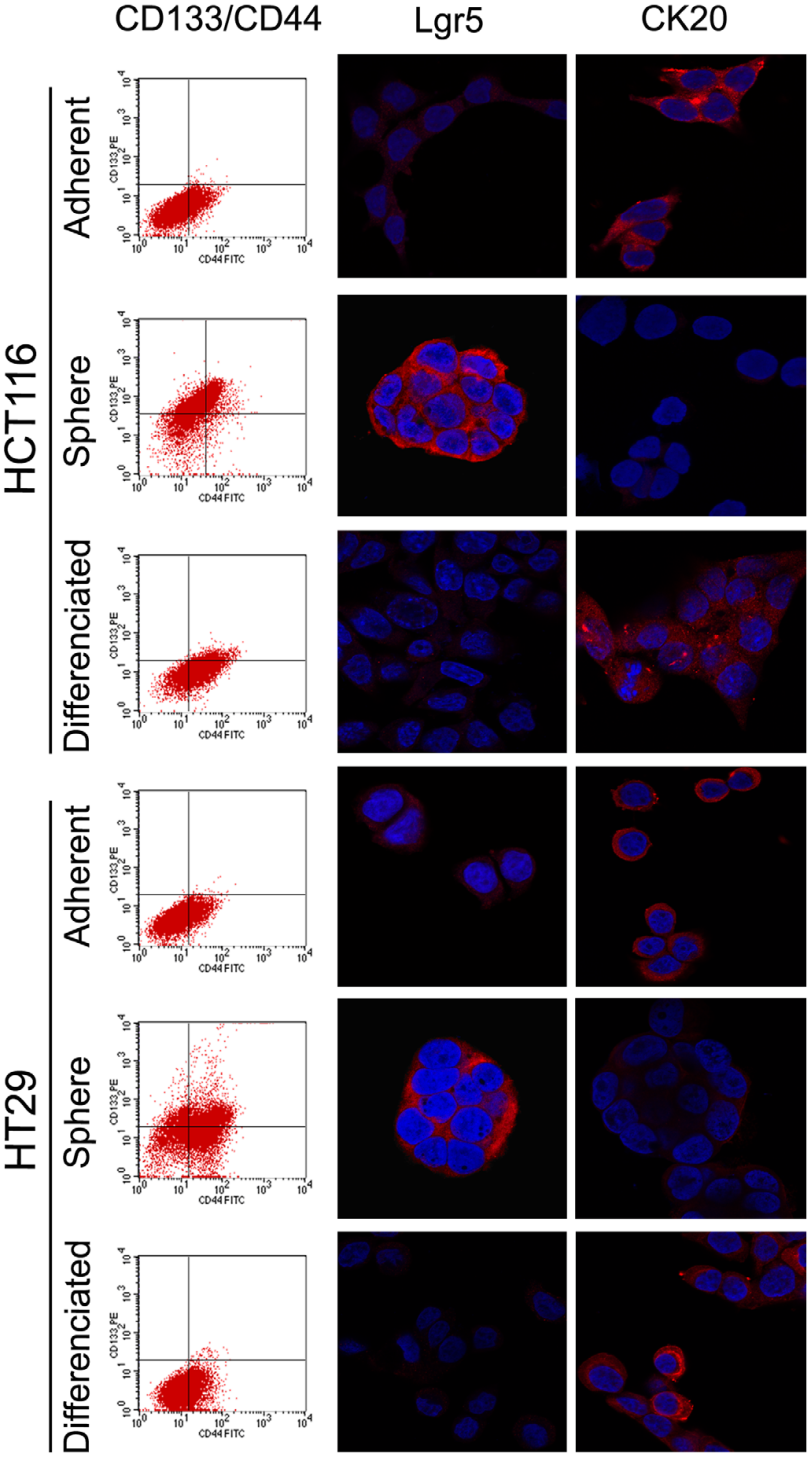
CD133/CD44, Lgr5, and CK20 expression of adherent, spheres, and differentiated HCT-116 and HT29. CD133 (known as cancer stem cell marker) expression rises from ≤1.2% up to 60%–80% when cultured in SFM, but reduced to 5%–10% when the colonsphere changed back into adherent cells. The same change of Lgr5 (known as crypt stem cell marker) expression can be found while CK20 expression, the marker of differentiated epithelial cell, showed the adverse change.

The spheroid cells showed higher levels of CD133 and Lgr5 (known as colon CSC markers), but lower levels of differentiated epithelial cell marker CK20 than the corresponding parental cells. The proportion of the CD133 positive cells was found to be less than 1% in parental cell lines, but more than 80% observed in spheroid cells (Figure 2). When the colonospheres were subjected to immunofluorescence staining for Lgr5, obvious Lgr5 staining was observed on the the surface of most spheroid cells indicating the presence of CSCs in colonosphere (Figure 2). When the spheroid cells were cultured in 10% FBS containing medium, they attached to the plastic, gradually migrated from colon spheres and changed into adherent cells morphologically. Flow cytometry and immunofluorescence examination showed down-regulated CD133 and Lgr5 expression with up-regulated CK20 after 4 weeks’ culturing (Figure 2). The results suggest that CSCs were efficiently enriched in spheroid cells, and they have the capacity of self-renew and multi-differentiation.

### Spheroid Cells Display Higher Capacity of Proliferation and Clone Formation

**Figure 3:**
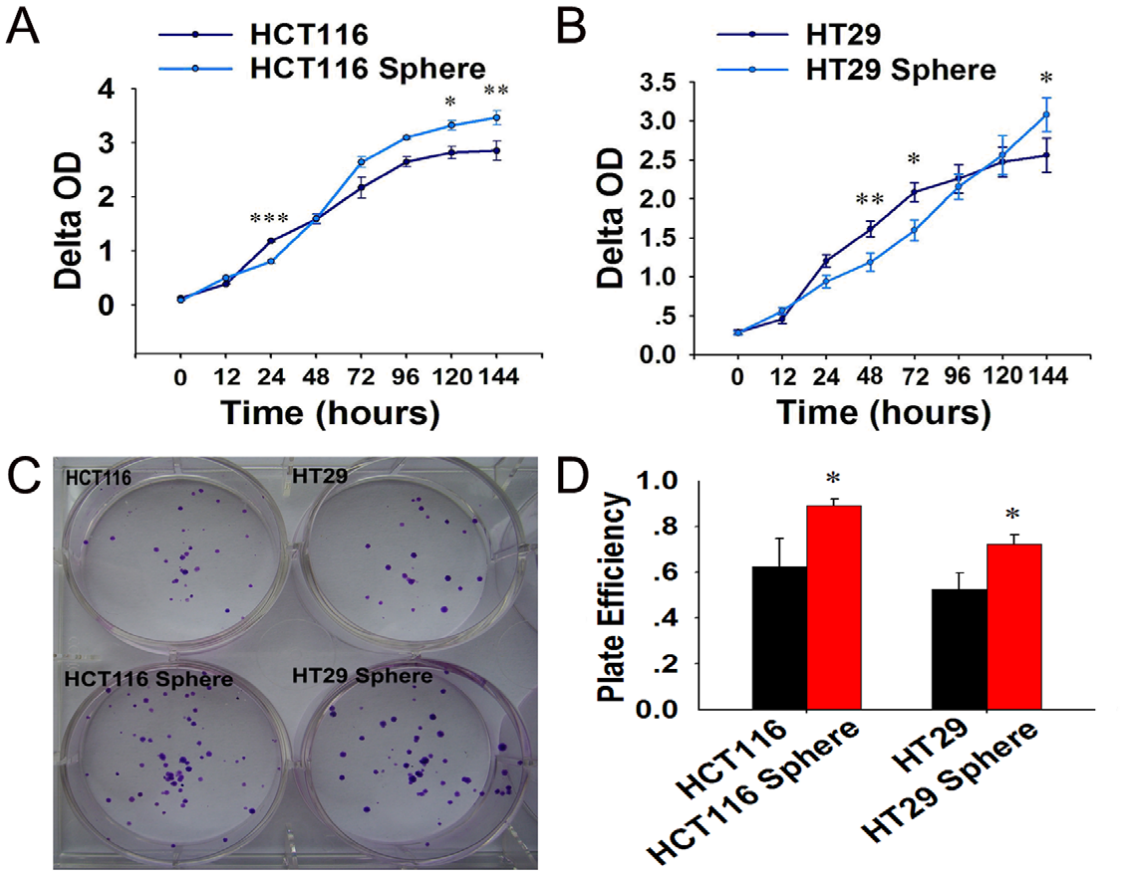
Proliferation and clone formation of adherent and spheroid cells of HCT-116 and HT29. (A–B) Proliferation of HCT116 and HT29 spheroid cells were higher than that of their parental counterparts after a period of adaptation to serum containing medium. (C–D) The capacity of colony formation in spheroid cells groups was higher than that of their parental counterpart.

We further determined whether the spheroid cells have capacity of higher cell proliferation using CCK-8 assay. The proliferations of spheroid cells were higher than that of their parental counterpart after a period of adaptation to serum containing medium (Figure 3A and 3B). And the capacity of colony formation in spheroid cells groups was higher than that of their parental counterpart (Figure 3C and 3D).

### Spheroid Cells Display Lower Adhesion but Higher *in vitro* Migratory/invasive Capacity

**Figure 4:**
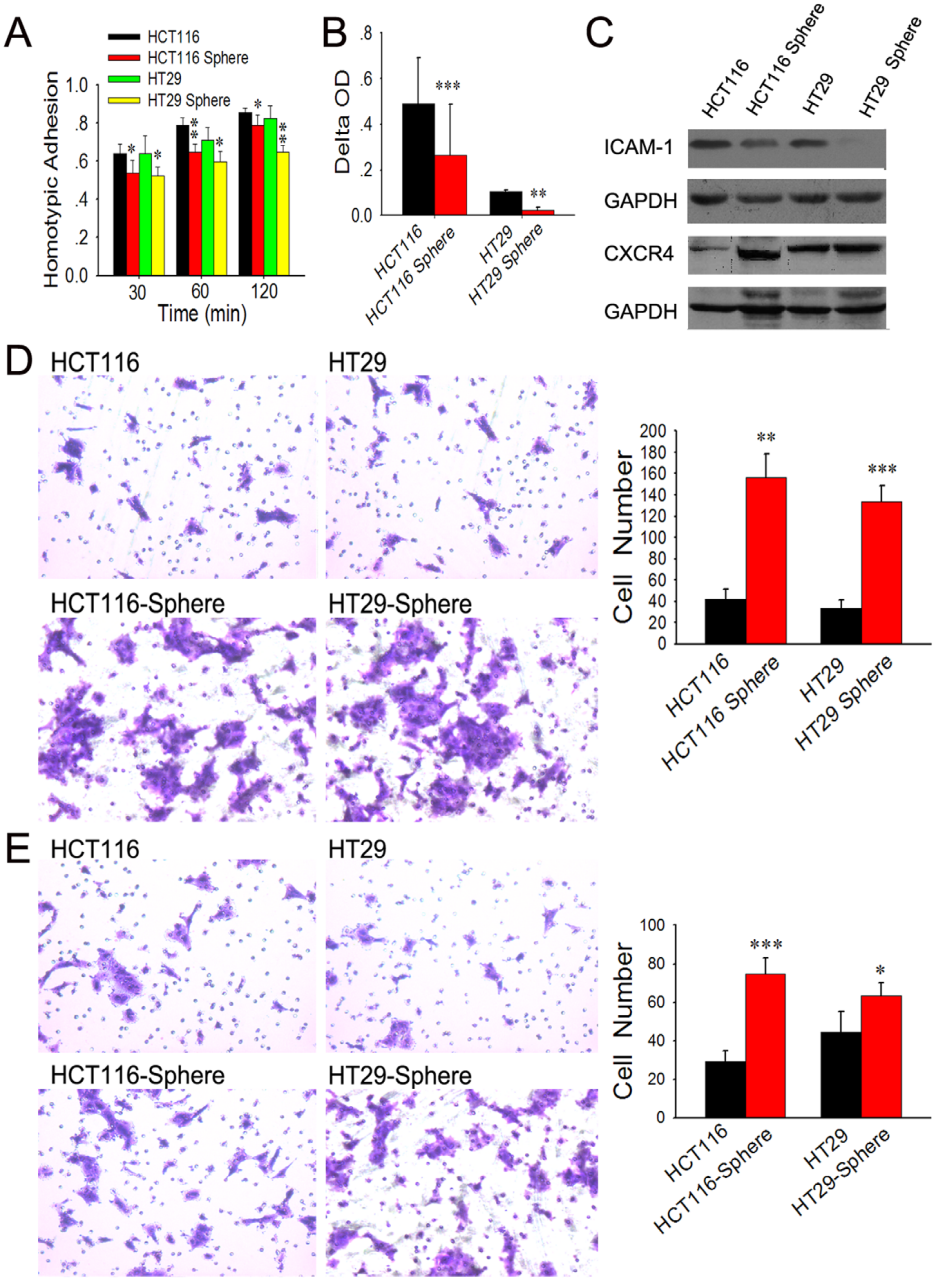
Cell adhesion and migratory/invasive capacity of adherent and spheroid cells. (A–C) Spheroid cells have lower capacities of both adhesions to FN (A) and to their homogeneous neighbors (B), as compared with their parental cells. The ICAM-1 expression in speroid cells was modestly down-regulated, while CXCR4 expression was up-regulated as compared to HCT116/HT29 monolayer cells respectively (C). (D and E) HCT116/HT29 spheroid cells showed a 3.8-fold (p<0.01) and 4-fold (p<0.001) increase in chemotactic potential at 24 h (D). More spheroid cells were obviously observed to invade Matrigel as compared to their parental counterparts (p<0.001 and p<0.05, respectively, E).

To investigate the malignant profile of spheroid cells, we conducted in vitro assays to evaluate the adhesion and migratory/invasive capacity of spheroid cells in comparison with their adherent counterparts. The results of cell adhesion assay demonstrated that spheroid cells have lower capacities of both adhesion to FN (one of the key ECM components) and adhesion to their homogeneous neighbors, as compared with their parental cells (Figure 4A and 4B). Then CXCR4 and ICAM-1 expression in HCT116/HT29 spheroid cells was examined and compared with their parental cells. The ICAM-1 level was modestly down-regulated, while CXCR4 expression was up-regulated in HCT116/HT29 spheroid cells when compared with HCT116/HT29 monolayer cells respectively (Figure 4C).

Using Transwell migration chambers, we found that spheroid cells display a significant increase in cell motility. Compared with HCT116/HT29 adherent cells, HCT116/HT29 spheroid cells showed a 3.8-fold (p<0.01) and 4-fold (p<0.001) increase in chemotactic potential at 24 h, respectively (Figure 4D). These cells also demonstrated higher capacity to invade Matrigel-coated inserts in Transwell migration chambers. More HCT116/HT29 spheroid cells were obviously observed to invade Matrigel as compared with corresponding adherent cells (p<0.001 and p<0.05, respectively) (Figure 4E). These results indicate that spheroid cells are endowed with lower adhesion, but higher migratory/invasive capacity, a functional phenotype associated with tumor aggressiveness.

### Spheroid Cells Promotes Proliferation, Migration and Tube Formation of EPCs Through Up-regulating VEGF Expression

**Figure 5:**
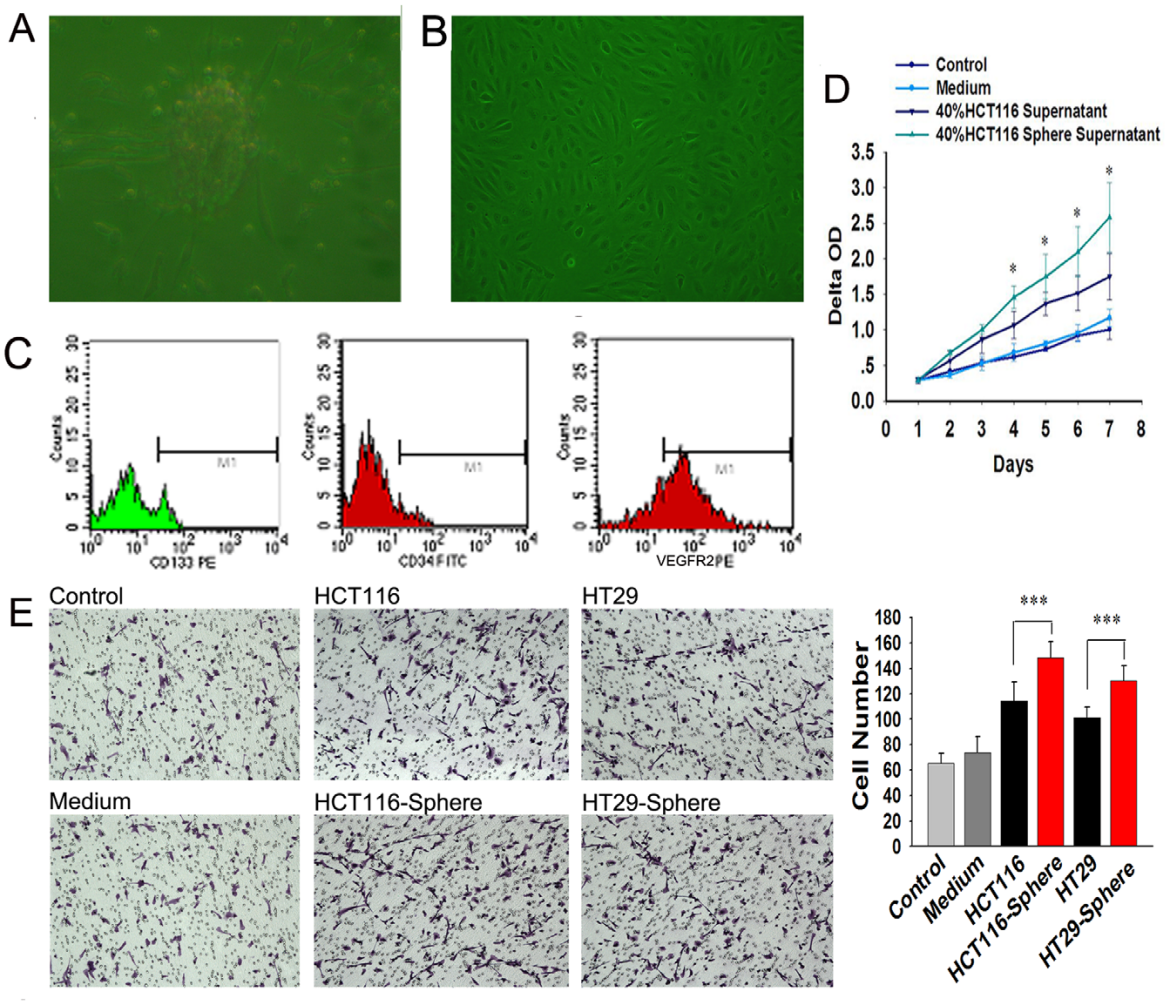
Supernatants of spheroid cells promote proliferation and migration of EPCs. (A-C) EPCs adhere to FN-coated flask after 72 hr and EPCs clones can be seen after one week (A), and present cobbles-like sign after two weeks (B). EPCs in later stage display a certain expression of CD34 and CD133, but high level expression of VEGFR2 (C). (D, E) Cell proliferation (D) and migration (E) of EPCs treated by supernatants of spheroid cells were higher than their counterpart.

**Figure 6:**
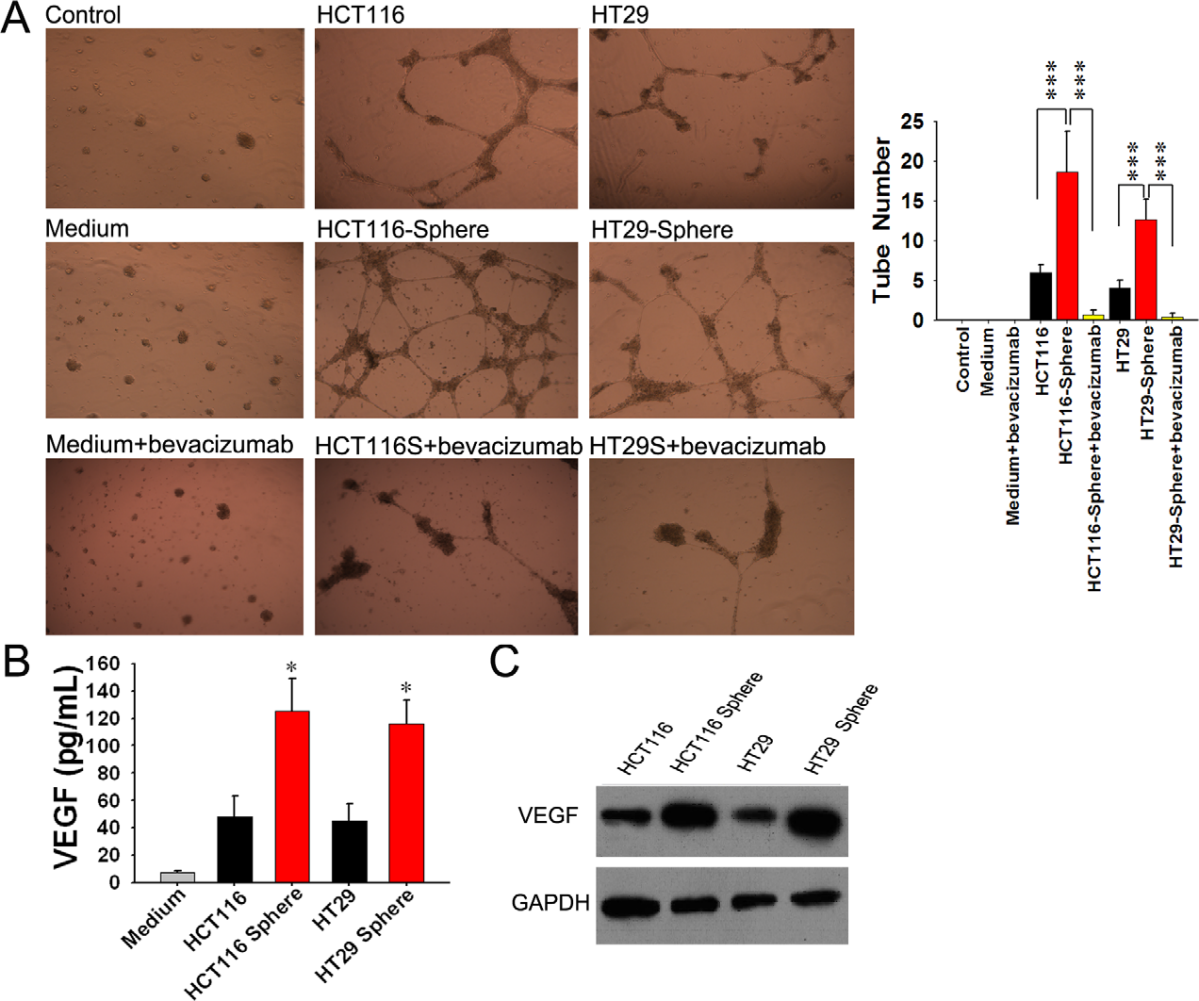
Supernatants of spheroid cells promote tube formation of EPCs through VEGF up-regulation. (A) Tube formation of EPCs treated by supernatants of spheroid cells was higher than their counterparts. And tube formation of EPCs was suppressed when we block the function of VEGF by bevacizumab. (B) The VEGF concentrations in supernatants of spheroid cells were higher than those of adherent cells. (C) Higher VEGF expression was observed in the spheroid cells compared with their counterparts.

Purified EPCs were obtained from human umbilical cord blood. Flow cytometry analysis showed that EPCs in later stage displayed a certain expression of CD34 and CD133, but high level expression of VEGFR2 (Figure 5A–5C). Cell proliferation and migration of EPCs induced by supernatants of spheroid cells were higher than their counterparts (Figure 5D and 5E). The tube formation assay showed that more tubes formed in spheroid cells group than adherent cells group. Additionally, when 0.25 mg/ml bevacizumab (avastin, a VEGF monoclonal antibody) was added in supernatant of spheroid cells, the tube formation capacity of EPCs was suppressed (Figure 6A). Then the VEGF secretions in supernatants of spheroid/adherent cells were detected using ELISA. The results showed that concentration of VEGF in supernatant of adherent cells was lower than spheroid cells (Figure 6B). The expression of spheroid/adherent cells were evaluated by Western blot (Figure 6C). The results illustrated that VEGF may play an important role in the effect of spheroid cells on EPCs, which suggested that spheroid cells may promote angiogenesis of EPCs through up- regulating VEGF expression.

### Spheroid Cells Possess Higher Tumorigenic and Metastatic Potential *in vivo* and EPCs Can Increase these Capacities

**Figure 7:**
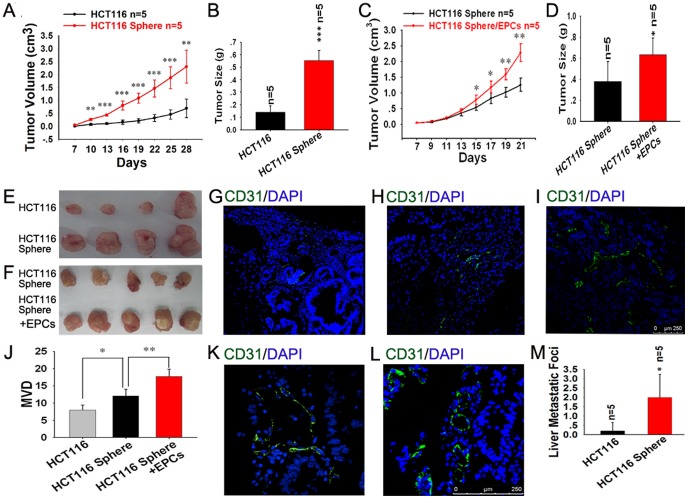
Spheroid cells possess higher tumorigenic and metastatic potential in vivo and EPCs can increase these capacities. (A–F) The spheroid cells showed significantly higher tumorigenecity than adherent cells (A, B, E). And the volumes of subcutaneous tumors in spheroid cells plus EPCs groups were larger than those of spheroid cells groups (C, D, F). (G–J) Microvessel density (MVD) of tumor developed by HCT116 spheroid cells (H) was higher than that of HCT116 (G). The highest MVD among these groups was found in tumor tissue developed from HCT116 sphere plus 1/10 EPCs (I, J). And more blood vessel developed from human EPCs in HCT116 sphere plus EPCs group (L) were found than that of pure spheroid cells group (K). (M) The HCT116 spheroid cells developed more liver metastatic foci than HCT116.

The spheroid cells showed significantly higher tumorigencity than adherent cells (Figure 7A, 7B and 7E). When 2×10^6^ cells were inoculated into nude mice, all mice developed tumors. Transplanted tumors were confirmed as colon cancers with hematoxylin-eosin staining. And the volumes of subcutaneous tumor in spheroid cells groups were significantly higher than that of adherent cells groups. Moreover, the volumes of subcutaneous tumor in spheroid cells plus 1/10 EPCs groups were higher than that of pure spheroid cells groups (Figure 7C, 7D and 7F). But no significant difference was found between pure spheroid cells and spheroid cells plus 1/100 EPCs (data not shown). Then blood vessels density in tumor tissues were evaluated using CD31 immunofluorescence staining. More blood vessels were found in spheroid cells group compared with adherent cells group, and even more angiogenesis in tumor tissue was observed in the combined group of HCT116 sphere plus 1/10 EPCs (Figure 7G–7J). And the MVD were counted according to the non-specific polyclonal CD31 antibody. In order to determine the EPCs contribution, we performed IF staining using human-specific monoclonal CD31 antibody. The result showed more blood vessels in the combined group of HCT116 sphere plus EPCs than pure spheroid cells (Figure 7K–L).

As the liver metastasis was concerned, four mice (4/5) developed liver metastasis in spheroid cells group, but only one mouse (1/5) did in adherent group. Metastatic tumors were also confirmed as colon cancers with hematoxylin-eosin staining (figure not shown). The result of liver metastatic foci counting showed the similar results (Figure 7M). The overall conditions of the mice in spheroid cells group are worse than those in adherent cells group, and some of them developed ascites and severe emaciation. And there were no significant difference between spheroid cells plus EPCs (with ratio of 10∶1 and 100∶1) groups and spheroid cells groups (data not shown).

### Clinical Evidence for Cancer Stem Cells Promote Angiogenesis in Human Colon Cancers

**Figure 8:**
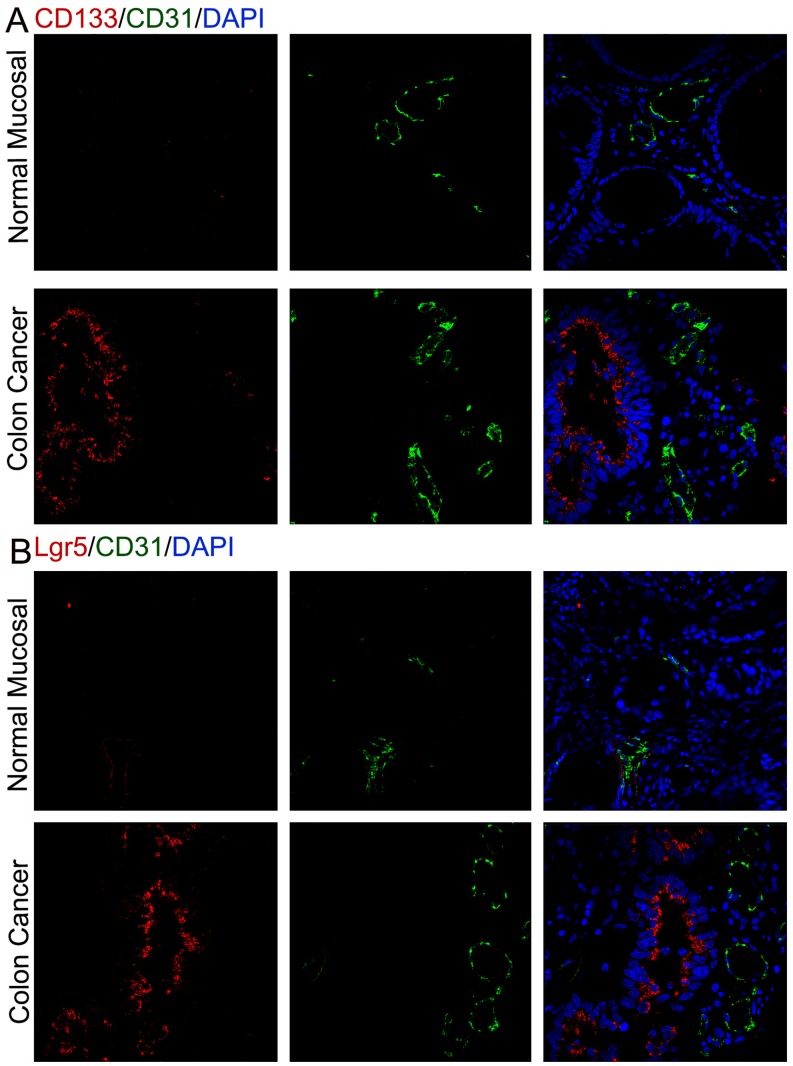
Cancer stem cells promote angiogenesis in human colon cancers. (A) More microvessels in cancer tissue than normal tissue were observed by immunofluorescent staining of CD133 and CD31 of normal mucosa and colon cancer. And higher MVD were found in the area enriching CD133^+^ cancer stem cells in colon cancer tissue. (B) Higher MVD (CD31^+^) were found in the area enriching Lgr5^+^ cancer stem cells in colon cancer tissue.

To support our experimental findings, we examined whether colon cancer stem cells promote angiogenesis within human colon cancers, and the normal mucosa from the same patient as control. The immunofluorescent staining of CD133/Lgr5 (colon cancer stem cell marker) and blood vessel EC marker CD31 were performed. As we all know, there are more microvessels in cancer tissue than normal tissue. In addition to this, we found that higher microvessel density in the area enriching cancer stem cells in the fresh specimen of human colon cancer (Figure 8).

## Discussion

In the 70s of the last century, Fidler IJ et al. [32] proposed the concept of tumor heterogeneity, which presume there were different phenotypes in the tumor tissue. The cancer stem cell theory was shaped based on this concept. Cancer stem cell is generally defined as a small sub-population of tumor cells bearing self-renewal and multi-directional differentiation potential [11]. Up to now, the existence of cancer stem cells has been confirmed in several solid tumors, including colon cancer. According to this hypothesis, the small subpopulation of cancer stem cells was bearing the whole characteristic of malignancy, including infinite proliferation, invasion and metastasis, relapse and resistance to therapy.

Nowadays, the extensively used method to isolate or enrich colon cancer stem cells is cell sorting based on cell surface markers, such as CD133, Musasai-1, CD44, EpCAM, Lgr5, etc. Ricci-Vitiani L et al. reported the CD133^+^ cells which accounts for about 2.5% of the tumour cells are tumorgenic cells in colon cancer [33]. Subcutaneous injection of colon cancer CD133^+^ cells readily reproduced the original tumor in immunodeficient mice, whereas CD133^−^ cells did not develop tumors. However, there were still many different opinions on these cancer stem cell markers. For example, Shmelkov SV proposed that CD133 expression is not restricted to stem cells, and both CD133^+^ and CD133^−^ metastatic colon cancer cells initiate tumors [12]. Some researchers reported the dye-effluxing side population cells expressing ABCG2, an ATP-binding cassette half-transporter, could be isolated as cancer stem cells [34], [35], [36]. However, several studies showed evidence against an association between the SP population and cancer stem cells. SP cells isolated from MKN28 gastric cancer cells did not produce tumor in a xenograft model [37]. Furthermore, SP and non-SP cells of colon cancer cell lines demonstrate equivalent multipotential differentiation capacity and similar tumorigenicity in xenograft model [38].

In the present study, we obtained tumor spheres from HCT116 and HT29 colon cancer cell lines by culturing these cells in serum-free medium [39], [40]. And the following analysis indicated that CD133 and Lgr5 expression of these spheroid cells was higher than their parental counterpart, while CK20 expression was lower which represents the differentiated endothelial cell. The contradictory phenotypes were detected when the spheroid cells were induced to differentiate in FBS containing medium. Moreover, the spheroid cells can form the same spheres, indicating that they have the self-renew capacity. From these results, we can draw the conclusion that cancer stem cells were enriched in these spheroid cells.

In order to take a further insight into the biological behavior of these spheroid cells, we performed a series of functional experiments. The results showed that spheroid cells display higher capacity of proliferation and clone formation, which represent self-renewal potential, as compared to their adherent counterparts. And also, spheroid cells demonstrate lower capacities of both adhesions to FN (one of the key ECM components) and adhesion to their homogeneous neighbors. That was to say, this kind of cells are easier to release from the bulk tumor. ICAM-1 is an important cell surface molecular mediating the intercellular adhesion and its downregulation can explain the lower homogenic adhesion of spheroid cells. Our results indicated that spheroid cells have enhanced in vitro migration and invasion potential. CXC chemokine receptor 4 (CXCR4) interacts specifically with the stromal cell-derived factor-1 (SDF-1), which is expressed in stromal cells, including fibroblasts and endothelial cells [41], [42]. Recent studies showed that chemotaxis effect of CXCR4/SDF-1 axis is related with lymph node and liver metastasis of CRC [43], [44], [45], [46]. And in our study, we found expression of CXCR4 of spheroid cells were upregulated.

The circulating, bone-marrow-derived EPCs contribute to angiogenesis- mediated pathological neovascularization, and recent studies have begun to recognize the biological significance of this contribution, especially to the growth and metastasis of tumors. Generally speaking, circulating EPCs recruit to sites of tumors following gradients of growth factors, such as vascular endothelial growth factor (VEGF), SDF-1 etc, which are released by tumor [47]. Kyrezek et al. reported the expressions of VEGF and SDF-1 in tumors could be enhanced each other. And in the other hand, VEGF could up-regulate the expression of CXCR4 in vascular endothelial cell, thus promote the migration of EPCs and contribute to tumor neovascularization [48]. In our study, we isolated EPCs, which display a certain expression of CD34 and CD133, but high level expression of VEGFR2 in its later stage. And we found that supernatants of spheroid cells can promote proliferation, migration and tube formation of EPCs by up-regulating VEGF expression and bevacizumab can suppress the angiogenesis of EPCs by blocking the function of VEGF (Figure 5, 6). The results of xenograft model indicated that EPCs can promote the growth of subcutaneous tumor of spheroid cells by mediating neovascularization (Figure 7). And using the human specific CD31 antibody, we can detect the blood vessel developed from EPCs. The results indicated that EPCs may play an important role in the neovascularization. It can be seen that VEGF is relevant to the interaction between EPCs and CSCs. Since the EPCs may be responsible for turning on the “angiogenic switch”, our results indicates a therapeutic strategy can be employed to keep this switch in the “off” position for colorectal cancer [47]. That is to say, if we block the interaction between EPCs and CSC, decreased tumor vascularization and suppressed tumor progression can be expected. Although no significant difference were found between spheroid cells plus EPCs groups and spheroid cells groups in liver metastasis, which may be due to the short observation period and limited sample size.

To further confirm the correlation between colon cancer stem cells and the tumor neovascularization, we then performed immunofluorescence in tumor tissues. CD31 expression is associated to CD133 or Lgr5 expression in colon cancer tissues (Figure 8), which indicates robust angiogenesis is stimulated by cancer stem cells. In recent years, the evidence strongly indicates that CSCs not only promote self-renewal and tumor initiation, but also induce and stimulate angiogenesis. They seem to be adept at initiating a potent angiogenic response by inducing and secreting certain proangiogenic factors, such as VEGF, which constitute one of the CSC factor subsets [40], [49]. These factors may promote the EPCs migration and neovascularization. Interestingly, in addition to the close link between angiogenesis and CSCs [50], certain stem-like cells can transdifferentiate into endothelial-like cells to form tumor blood vessels via a process termed vasculogenic mimicry [51], [52], [53]. This can be used to explain why human CD31 antigen exists in subcutaneous tumor developed from spheroid cells (Figure 7K).

Taken together, our results demonstrate that HCT116 and HT29 spheroid-derived cells possess the characteristics of cancer stem cells, including higher capacity of proliferation and clone formation, higher migratory and invasive potential, and also enhanced tumorigenic and metastatic potential. The spheroid cells could promote the proliferation, migration and tube formation of EPCs through secretion of VEGF, and simultaneously EPCs could increase tumorigenic capacity of spheroid cells through angiogenesis vice versa. Our findings indicate that the coaction of CSCs and EPCs may play an important role in the development of colon cancer.
